# Supplementary figures and images for: EEG-based characterization of auditory attention and meditation: an ERP and machine learning approach
Source: Front Hum Neurosci. 2025 Aug 26;19:1616456. doi: 10.3389/fnhum.2025.1616456 (PMC12417730; doi:10.3389/fnhum.2025.1616456)

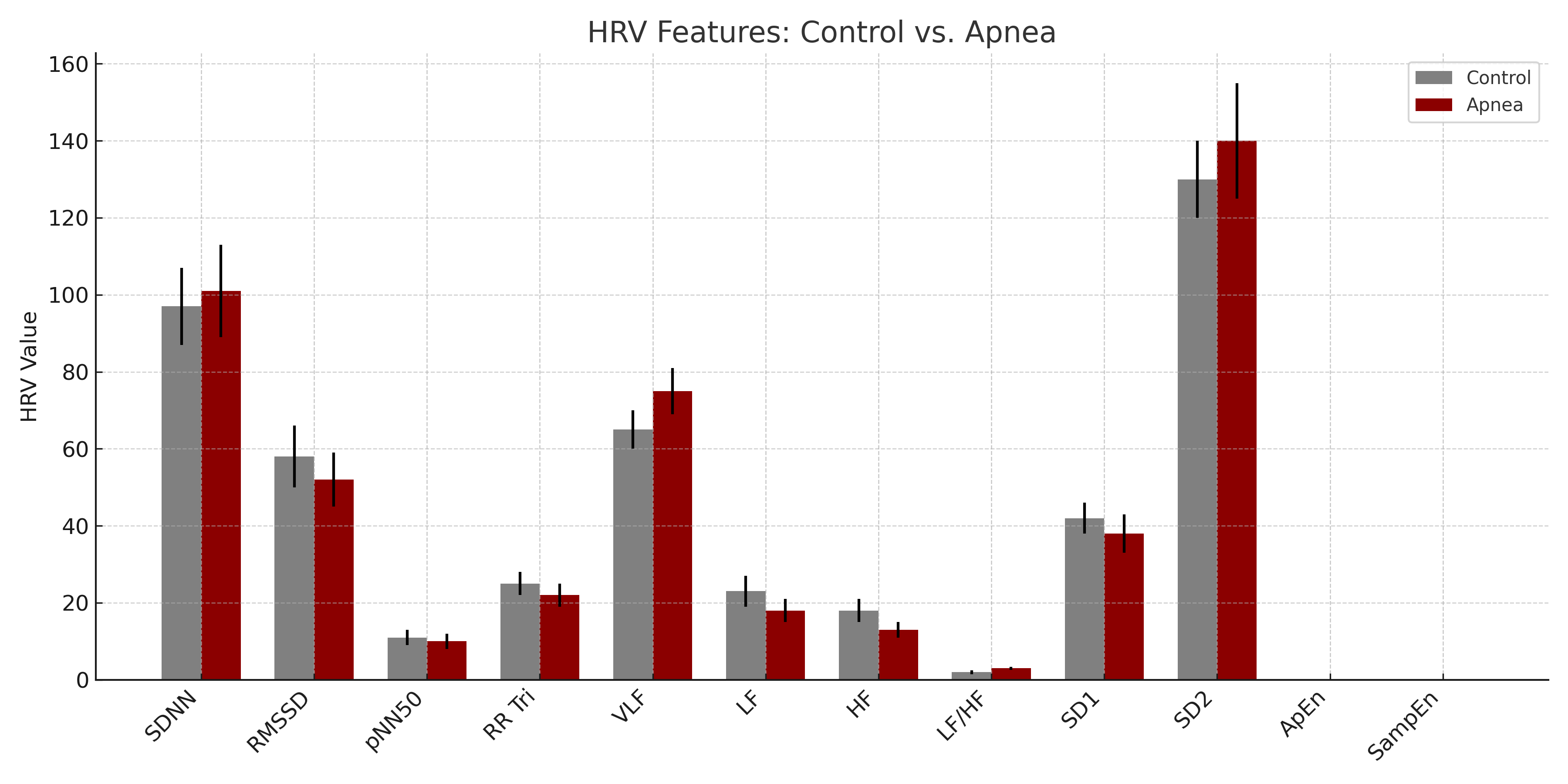

Supplement: Supplementary file 1 [file Image_1.png]

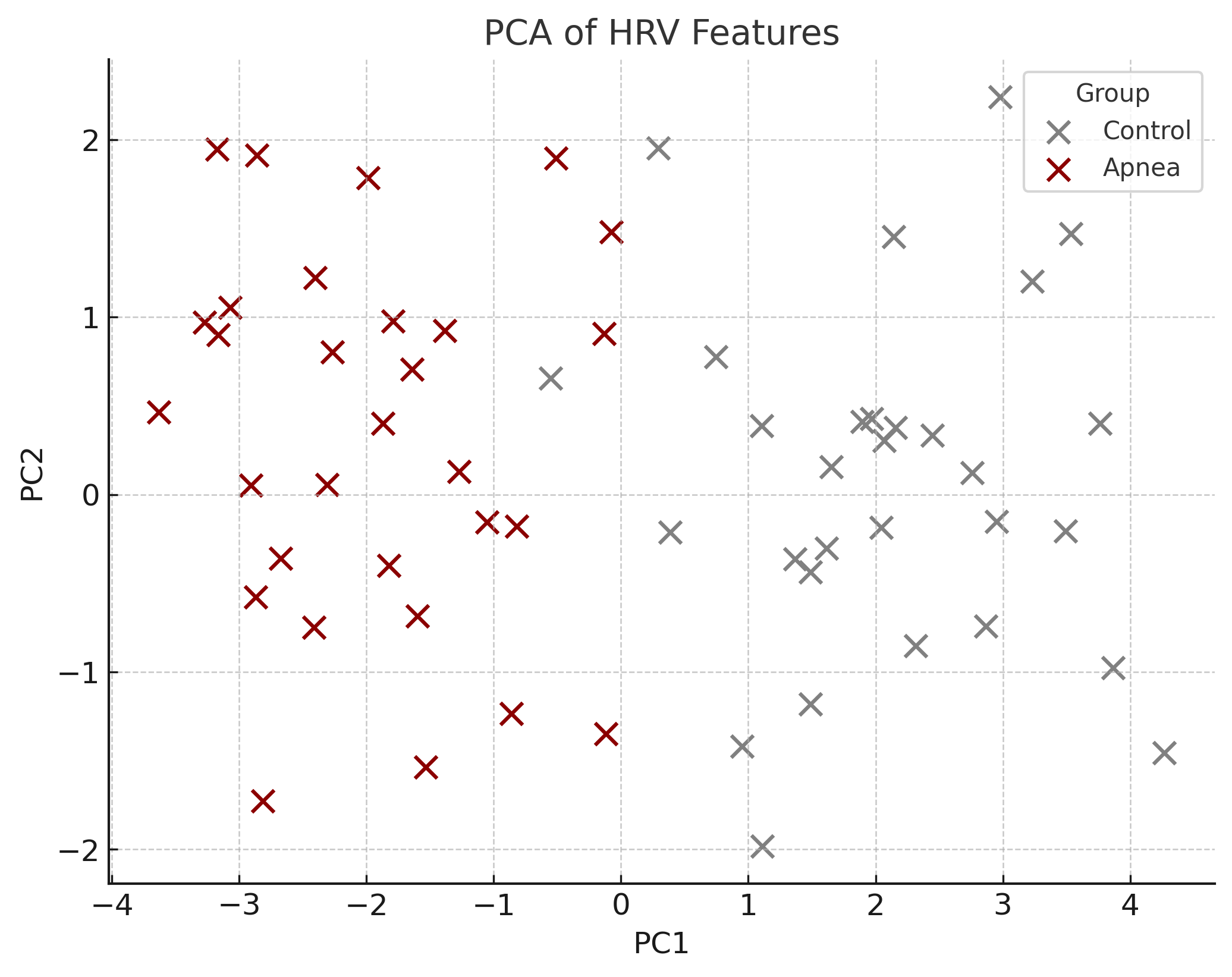

Supplement: Supplementary file 2 [file Image_2.png]

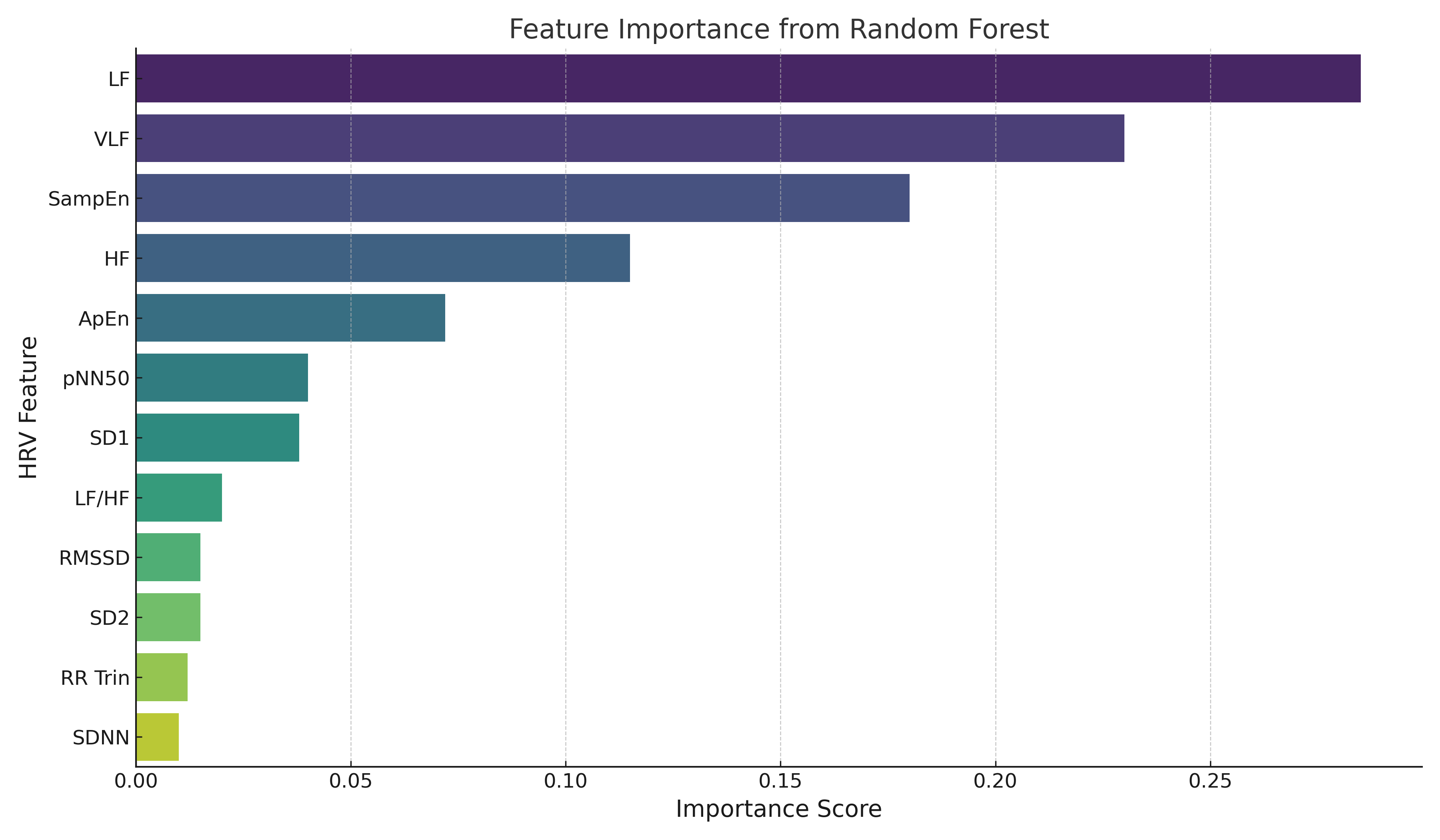

Supplement: Supplementary file 3 [file Image_3.png]

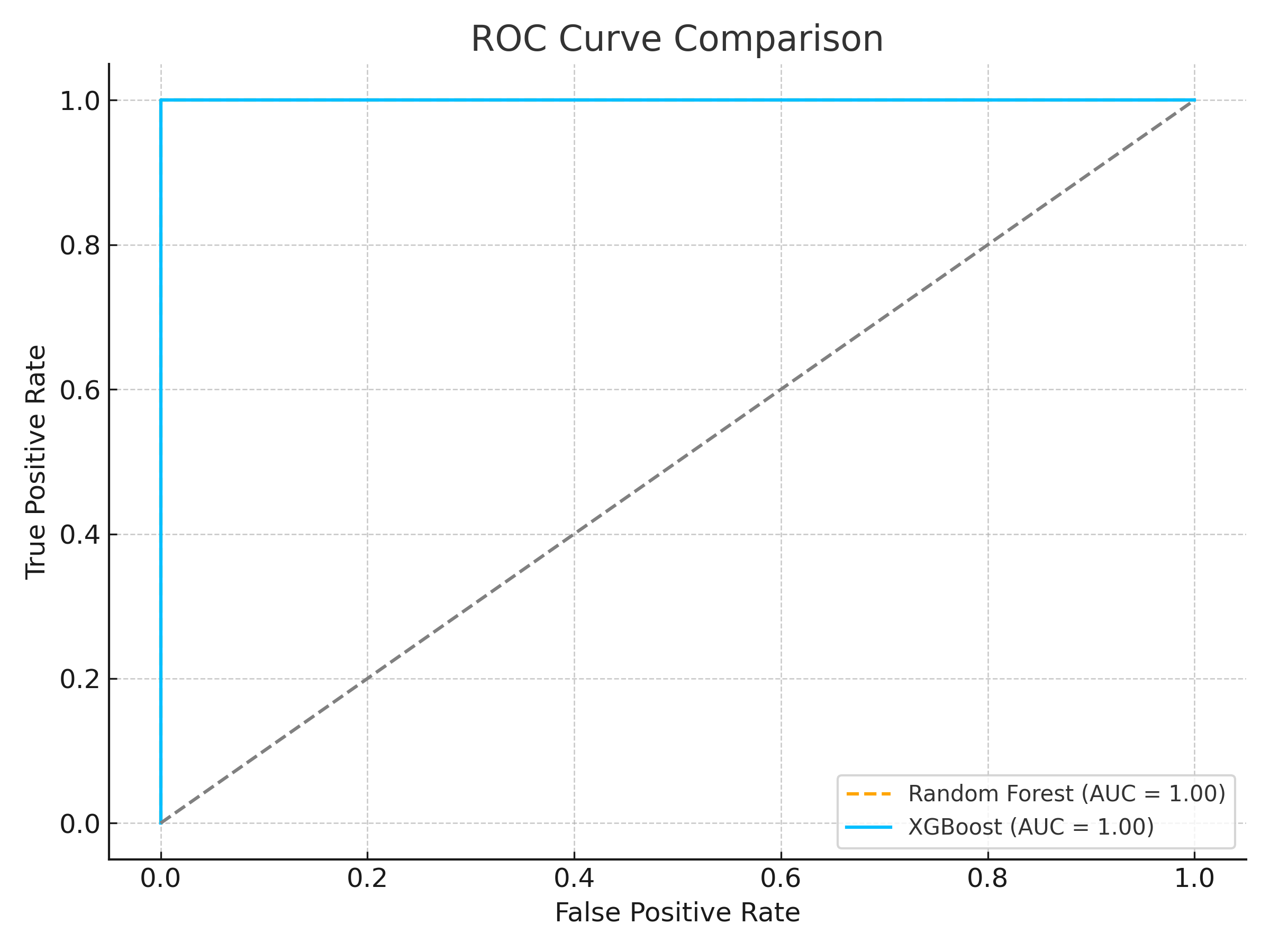

Supplement: Supplementary file 4 [file Image_4.png]

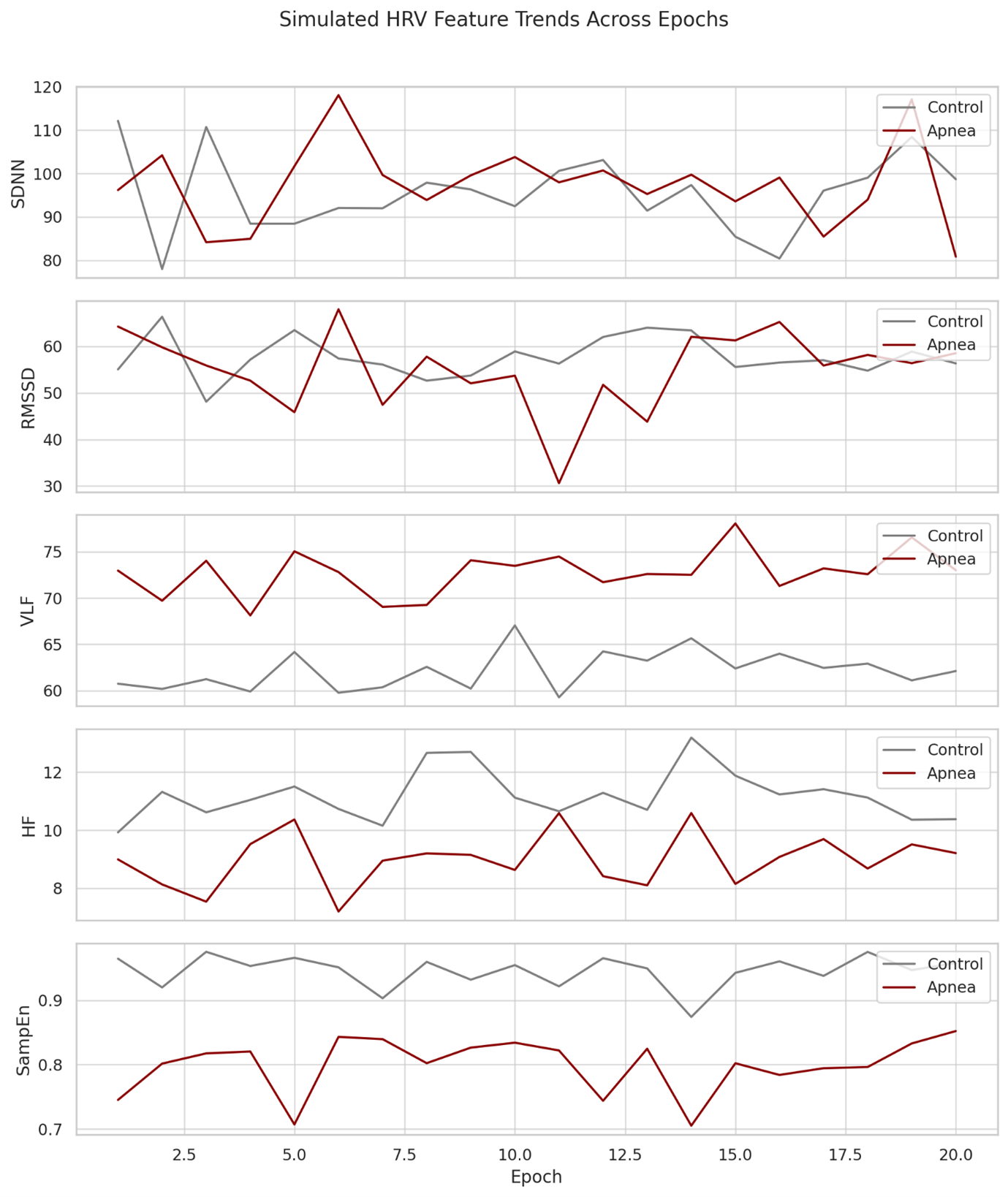

Supplement: Supplementary file 8 [file Image_8.png]
